# Intensive Chemotherapy Versus Venetoclax-Based Regimens in Elderly Patients with Acute Myeloid Leukemia: Is the Chemotherapy Era Ending?

**DOI:** 10.3390/jcm14082759

**Published:** 2025-04-17

**Authors:** Mirko Farina, Michele Malagola, Simona Bernardi, Federica Re, Domenico Russo, Daniele Avenoso

**Affiliations:** Unit of Blood Diseases and Bone Marrow Transplantation, Department of Clinical and Experimental Science, University of Brescia, ASST Spedali Civili di Brescia, 25123 Brescia, Italy; mirko.farina@unibs.it (M.F.); michele.malagola@unibs.it (M.M.); simona.bernardi@unibs.it (S.B.); federica.re@unibs.it (F.R.); domenica.russo@unibs.it (D.R.)

**Keywords:** AML, allo-HSCT, non-intensive chemotherapy, venetoclax-based regimens

## Abstract

**Background:** Acute myeloid leukemia (AML) primarily affects older adults and is associated with poor prognosis, particularly in patients aged ≥ 60 years with comorbidities and adverse disease characteristics. Standard intensive chemotherapy, such as the “7 + 3” regimen, has shown limited efficacy and substantial toxicity in this population, underscoring the need for alternative treatment strategies. In recent years, venetoclax-based regimens have emerged as an important option, demonstrating promising outcomes in elderly patients traditionally considered unfit for intensive therapy and, more recently, even in selected fit patients. **Methods:** This narrative review provides a comprehensive comparative analysis of intensive chemotherapy and venetoclax-based regimens in elderly AML patients. This review synthesizes evidence from prospective and retrospective clinical trials, with focuses on treatment efficacy, safety, and the ability to bridge patients to curative allogeneic hematopoietic stem cell transplantation (allo-HSCT). **Results:** Intensive chemotherapy has achieved complete remission (CR) rates of 40–60% in elderly AML patients, though the median overall survival (OS) rarely exceeds 12 months. Conversely, venetoclax combined with hypomethylating agents has recently demonstrated CR rates of up to 74%, with 83% of responders proceeding to allo-HSCT in selected studies. Venetoclax-based regimens have also been associated with improved tolerability and reduced treatment-related mortality. **Discussion:** This review highlights a paradigm shift in the management of AML in the elderly. While intensive chemotherapy remains a standard option for selected patients, the increasing use of venetoclax-based regimens represents a novel and effective strategy with the potential to overcome traditional limitations, especially in patients previously deemed ineligible for curative approaches. The high remission and transplantation rates observed with non-intensive therapies support their role not only as a palliative alternative but as a bridge to cure. **Conclusions:** Venetoclax-based regimens are reshaping the treatment landscape of AML in the elderly, offering high response rates and facilitating access to allo-HSCT. Further research is needed to optimize treatment sequencing, explore novel combinations, and reduce relapse rates after transplants, ultimately improving the long-term outcomes in this high-risk population.

## 1. Introduction

Acute myeloid leukemia (AML) is a heterogeneous hematologic malignancy characterized by the clonal proliferation of immature myeloid cells within the bone marrow causing disruption of normal hematopoiesis, leading to cytopenias, life-threatening infections, and bleeding complications [1,2]. With a median age of diagnosis of 67 years, AML is mainly diagnosed in older adults, and its incidence increases with age [3]. Despite advancements in understanding its pathogenesis, risk stratification, and treatment, AML remains a challenging disease to treat, particularly in the elderly (≥60 years) population [4]. Over the last 50 years, the treatment landscape for AML has undergone significant evolution.

Intensive chemotherapy has been the backbone of AML treatment for several decades, primarily aimed at achieving complete remission (CR) through the rapid eradication of leukemic blasts. The standard protocol, commonly known as the “7 + 3” regimen, combines seven days of continuous infusion of cytarabine with three days of an anthracycline such as daunorubicin or idarubicin. This approach, initially developed in the 1970s, transformed AML treatment, demonstrating high efficacy in achieving CR in younger patients. However, its application in elderly populations presents unique challenges and limitations. In elderly patients, intensive chemotherapy is often associated with diminished outcomes due to a combination of age-related physiological decline and disease-related factors. Studies consistently report CR rates ranging from 40% to 60% in this population, significantly lower than those observed in younger cohorts. Furthermore, the median overall survival (OS) for elderly patients undergoing intensive chemotherapy rarely exceeds 12 months, with a substantial proportion of patients succumbing to treatment-related complications or early relapse. The biological characteristics of AML in elderly patients also play a significant role in the reduced efficacy of intensive chemotherapy [5]. Older patients are more likely to present with adverse cytogenetic and molecular profiles, such as complex karyotypes, TP53 mutations, and secondary AML (s-AML). These features are strongly associated with resistance to chemotherapy and higher rates of relapse, further limiting the utility of standard intensive regimens in this demographic and therefore the access to curative allo-HSCT.

Toxicity is a critical concern when considering intensive chemotherapy for elderly AML patients. Regardless of the specific type of intensive induction chemotherapy, this strategy is linked with significant adverse events such as neutropenic fever, mucosity, cardio-toxicity, and renal and hepatic disfunction. These are likely the reasons for the same overall survival of AML patients if they are receiving standard 7 + 3 or high-dose cytarabine regimens like FLAG-IDA; the toxicity “fee” for achieving complete remission is paid with life-threatening or lethal toxicities that can preclude further cycles of treatment and access to allo-HSCT, therefore exposing the patients to a very high risk of disease relapse. Ultimately, these severe side effects are the elements for the onset of treatment-related mortality; in elderly patients undergoing intensive chemotherapy, it ranges from 10% to 25% in the first 30 days of treatment, driven primarily by infectious complications and cardio-renal failure [6,7].

During the 1980s and 1990s, the advent of allogeneic hematopoietic stem cell transplantation (allo-HSCT) introduced a potentially curative strategy, particularly for high-risk patients [8,9]. Supportive care measures, including transfusions, antibiotics, and growth factors, alongside the availability of reduced intensity conditioning regiments, further improved survival rates during this era, allowing more AML patients access to this procedure [8,9,10].

The early 2000s saw the development of targeted therapies, including FLT3 inhibitors [10] and IDH1/IDH2 inhibitors [11], aiming to either improve the efficacy of the 7 + 3 backbone or offer target therapy to elderly AML patients with comorbidities. The last decade has been characterized by the availability of the BCL-2 inhibitor venetoclax [12], which has been combined with several drugs aiming to improve the response rate in AML in the elderly and unfit. It has been used in combination with hypomethylating agents (HMAs) very frequently, representing a paradigm shift in AML treatment, particularly for patients ineligible for intensive chemotherapy.

In elderly (≥60 years) AML patients, treatment decisions are particularly complex. These patients often present with comorbidities, reduced physiological reserves, and adverse disease biology, including secondary AML and unfavorable cytogenetics. The median overall survival (OS) for elderly patients treated with intensive chemotherapy rarely exceeds one year, and treatment-related mortality is significant [13]. For many, standard approaches are either intolerable or ineffective, underscoring the need for alternative therapeutic strategies [14].

Also, in the last twenty years, it has become clear that on one hand, AML patients may need different treatment approaches (based on biology, commodity, and disease risk), and on the other hand, the use of allo-HSCT has become the primary endpoint of any AML curative treatment program, and it progressively moved from the second-line salvage therapy to the first-line intensification therapy for high- to intermediate-risk AML for younger to older patients of up to 75 years.

Previously, a randomized clinical study highlighted the beneficial effects of consolidation with allo-HSCT in high-risk disease. In the last two decades, the availability of reduced-intensity conditioning regimens and of alternative donor platforms has allowed more patients to be consolidated with allo-HSCT. However, the proportion of elderly patients undergoing allo-HSCT following intensive chemotherapy remains disappointingly low [15,16]. Data from clinical trials indicate that fewer than 20% of elderly patients transition to allo-HSCT, primarily due to treatment-related morbidity and mortality (impairing patients’ fitness for transplantation) and disease progression or refractoriness [6]. This is due to the low rate of complete remission achievable with intensive chemotherapy and the low clinical fitness shown from these patients after chemo-induction/consolidation treatment [17].

In elderly AML patients, non-intensive chemotherapy strategies have emerged as vital options for those unfit to tolerate intensive regimens due to age-related physiological decline or comorbidities [18]. These approaches aim to balance efficacy and tolerability, with the primary goal of achieving remission or stable disease while minimizing toxicity. Over the past two decades, low-dose cytarabine, hypomethylating agents (HMAs), and venetoclax-based regimens have significantly transformed the treatment landscape for this patient population. Low-dose cytarabine (LDAC) was among the first non-intensive approaches explored for elderly AML patients. Administered at subtherapeutic doses (20 mg/m² twice daily for 10 days per 28-day cycle), LDAC demonstrated modest efficacy in early clinical studies. Hypomethylating agents, including decitabine and azacitidine, represent a cornerstone of non-intensive therapy in AML. These drugs modulate DNA methylation, reactivating tumor suppressor genes and promoting the differentiation of leukemic cells. Both agents have shown superior outcomes compared to conventional care regimens in elderly AML patients.

Venetoclax, a selective BCL-2 inhibitor, has revolutionized AML treatment, especially in elderly patients unfit for intensive chemotherapy. BCL-2 overexpression in AML cells promotes resistance to apoptosis, and venetoclax effectively restores this apoptotic pathway. This review evaluates the role of intensive chemotherapy and non-intensive venetoclax-based regimens in this population, with a focus on outcomes from key clinical trials that are the fundamental instruments for daily clinical decisions.

## 2. Methods

This work is a narrative comparative review aimed at summarizing and analyzing the available evidence on intensive chemotherapy versus venetoclax-based regimens in elderly patients (≥60 years) with acute myeloid leukemia (AML). This review focuses on clinical outcomes, remission rates, and potential transition to allogeneic hematopoietic stem cell transplantation (allo-HSCT), drawing from key prospective and retrospective studies.

### 2.1. Eligibility Criteria

Studies were selected according to the following criteria:

- Population: Adult patients aged ≥60 years with confirmed diagnosis of AML;
- Interventions: Standard intensive chemotherapy regimens (e.g., “7 + 3” protocols) or non-intensive regimens based on venetoclax, often in combination with hypomethylating agents (HMAs) or low-dose cytarabine (LDAC);
- Outcomes: At least one of the following: overall survival (OS), complete remission (CR/CRi), event-free survival (EFS), or good rate of allo-HSCT;
- Study Design: Phase II–III clinical trials or retrospective cohort studies;
- Language: English;
- Publication Type: Peer-reviewed articles published between 2000 and 2024.

### 2.2. Information Sources and Search Strategy

We searched the literature using the PubMed, Embase, and Cochrane Library databases. The last search was conducted on December 2024.

The search strategy combined terms such as “acute myeloid leukemia”, “AML”, “elderly”, “older adults”, “venetoclax”, “hypomethylating agents”, “intensive chemotherapy”, “low-dose cytarabine”, “allo-HSCT”, and “transplantation”.

Reference lists of included studies and relevant reviews were manually screened to identify additional sources.

### 2.3. Study Selection and Data Extraction

Two authors independently screened titles and abstracts for eligibility, and full-text articles were retrieved for further evaluation. Discrepancies were resolved through consensus.

Key data were extracted and summarized, including the following:

- Study design and year of publication;
- Sample size and median age;
- Treatment regimens (intensive or venetoclax-based);
- Primary outcomes (OS, CR, EFS, allo-HSCT rates);
- Key safety or tolerability findings, when available.

### 2.4. Quality and Bias Considerations

Given the narrative nature of this review, no formal risk of bias scoring was performed. However, the study design, cohort size, and potential confounding factors were taken into account when interpreting and comparing the results.

## 3. Results

### 3.1. Intensive Chemotherapy

Several landmark clinical trials have evaluated the efficacy and safety of intensive chemotherapy in elderly AML patients, including trials conducted by the GIMEMA, EORTC, US Intergroup, and MRC groups. Those studies have provided valuable insights into the role of intensive chemotherapy in this population. The GIMEMA AML-15 trial evaluated the feasibility of adding gentuzumab ozagamicin (GO) to a modified 7 + 3 backbone that consisted of mitoxantrone, etoposide, and infusion cytarabine (MICE) for AML patients aged 61–75. While the combination of GO with MICE was tolerated, the CR+CR with incomplete recovery (CRi) rate was modest (50%), with a median overall survival (OS) of under one year, and that study highlighted the limited applicability of intensive chemotherapy in older patients with high-risk features [19]. Also, it has been shown that intensive chemotherapy associated with autologous stem cell transplantation as a consolidation strategy did not improve the clinical outcomes achieved with AML-15 [20]. The US Intergroup Study (CALGB 9720) explored the role of standard-dose chemotherapy in elderly AML patients, followed by IL-2 maintenance for those in CR. Despite a CR rate of 50%, the maintenance strategy was ineffective, with a median OS of 14 months in both the experimental and standard arms. Those studies have highlighted that despite the achievement of CR in nearly half of the subjects, disease relapse occurred early, and the lack of access to allo-HSCT was the main factor for a short OS [21]. The MRC AML11 showed that an intensified 7 + 3 backbone with three agents and multiple rounds of induction treatments were not able to improve the 5-year OS, which was nearly 10%. Interestingly, that was an attempt to improve the chemotherapy outcome without offering allo-HSCT to elderly AML patients [22]. Those prospective studies, despite different combinations of drugs within the induction phase, showed similar OSs, highlighting an unmet need for the consolidation of AML in the elderly in CR.

### 3.2. Non-Intensive Chemotherapy Strategies and Venetoclax-Based Regimens

The NCRI AML 14 trial compared LDAC to hydroxyurea or supportive care in elderly AML patients; the LDAC achieved higher response rates (CR: 18%) compared to the hydroxyurea (CR: 1%). However, the median overall survival (OS) remained limited (4–6 months), highlighting the suboptimal efficacy of LDAC monotherapy in this high-risk population [23]. LDAC has been combined with agents such as venetoclax or glasdegib in recent studies, showing improved response rates and survival outcomes compared with LDAC alone [24,25].

In the DACO-016 study, decitabine achieved a higher CR rate (17.8%) and prolonged median OS (7.7 months vs. 5.0 months) compared to LDAC or the best supportive care; this agent can be an option for elderly patients with intermediate-risk disease or those ineligible for novel targeted therapies [26]. Another hypomethilating agent, azacitidine, was evaluated in a similar way within the AZA-AML-001 study; the azacitidine improved the median OS (10.4 months vs. 6.5 months) and was associated with higher CR and partial remission rates [27]. Similar results for both hypomethilating agents are also associated with a favorable safety profile with a low incidence of treatment-related mortality.

Venetoclax, a selective BCL-2 inhibitor, has revolutionized AML treatment, especially in elderly patients unfit for intensive chemotherapy. BCL-2 overexpression in AML cells promotes resistance to apoptosis, and venetoclax effectively restores this apoptotic pathway. When combined with HMAs or low-dose cytarabine, venetoclax-based regimens have demonstrated impressive efficacy in clinical trials. The Phase III VIALE-A study was designed to evaluate the efficacy of venetoclax in combination with azacitidine compared with azacitidine alone in newly diagnosed elderly patients with acute myeloid leukemia (AML) who were unfit for intensive chemotherapy. That study demonstrated that the combination therapy significantly improved outcomes, achieving complete remission (CR) or complete remission with incomplete hematologic recovery (CRi) rates of 66.4% compared with the 28.3% for azacitidine alone. The median overall survival (OS) also showed substantial improvement, with the combination therapy yielding a median OS of 14.7 months versus 9.6 months with azacitidine alone. In terms of safety, the most common adverse events reported were febrile neutropenia, occurring in 42% of patients, and gastrointestinal symptoms such as nausea and diarrhea [28]. However, these adverse events were generally manageable with supportive care, allowing most patients to continue treatment. Similarly, the Phase III VIALE-C study assessed the efficacy of venetoclax combined with low-dose cytarabine (LDAC) compared with LDAC alone in elderly AML patients. That study found that the combination therapy achieved a significantly higher CR/CRi rate of 48% compared with only 13% with the LDAC alone. The median OS was also improved, with the combination therapy resulting in a median OS of 7.2 months compared with 4.1 months for the LDAC alone. Those studies collectively demonstrate the clinical benefits of venetoclax-based combination therapies in elderly AML patients. They highlight significant improvements in response rates and overall survival compared to standard treatments, with a manageable safety profile. Those studies firmly establish venetoclax-based regimens as a standard of care for elderly AML patients, offering a balance of high efficacy and manageable toxicity. The VEN-DEC trial specifically evaluated the combination of venetoclax and decitabine in elderly AML patients, with a focus on its role as a bridge to allogeneic hematopoietic stem cell transplantation (allo-HSCT). That multicenter phase II study enrolled patients aged 60–75 years, with intermediate- to high-risk AML, who were deemed unfit for intensive chemotherapy. The findings from that trial highlight the significant potential of venetoclax-based regimens in improving outcomes for this challenging patient population. The VEN-DEC regimen demonstrated remarkable efficacy, achieving a complete remission (CR) rate of 74%, with an additional 10% of patients achieving complete remission with incomplete hematologic recovery (CRi). Notably, 83% of the patients who achieved remission proceeded to allo-HSCT during their first remission [29]. This is a substantially higher proportion than typically observed with intensive chemotherapy and underscores the regimen’s potential to serve as a bridge to curative transplantation.

In terms of safety, the VEN-DEC regimen was generally well-tolerated. The most common adverse events were hematological, including neutropenia and thrombocytopenia. The non-hematological complications primarily involved febrile episodes, which were manageable within the scope of supportive care. This favorable safety profile makes VEN-DEC an attractive option for elderly patients who are traditionally more vulnerable to treatment-related complications. The results of the VEN-DEC study have important clinical implications. The combination of venetoclax and decitabine not only achieved high remission rates but also enabled a significant proportion of elderly patients to undergo potentially curative allo-HSCT. This represents a paradigm shift in the management of AML in this demographic, offering new hope for improved survival and long-term outcomes. The VEN-DEC regimen establishes venetoclax-based therapies as a promising strategy for bridging elderly AML patients to transplantation and enhancing their overall prognosis.

### 3.3. Transition to Allogeneic Stem Cell Transplantation (Allo-HSCT)

The HOVON-SAKK collaborative study group evaluated the outcomes of AML patients enrolled in three successive studies, and a reduced relapse rate for patients undergoing allo-HSCT in the first CR (CR1) from HLA-identical sibling donors was evident. Despite an increased risk of treatment-related mortality for the patients who had undergone allo-HSCT, there was still an advance in disease free survival when the allografted patients were compared with those without donors available; this also translated to a significant benefit of 12% in OS by donor availability for all AML patients allografted in CR1 without favorable cytogenetic profiles [30].

In 2020, Gardin and colleagues reported a complete remission rate of 72% but an allogeneic HSCT rate of 18% [31]. The access to allogeneic HSCT still remains a challenge for patients older than 60 years with acute myeloid leukemia. Less than 10% of these patients will undergo allogeneic HSCT due to several factors, such as an inadequate complete response to induction, therapy-related toxic effects, comorbidities, or delayed referral to transplant centers. While intensive chemotherapy is considered an option for select elderly patients, its limitations in bridging elderly patients to allo-HSCT (shown in Table 1) underscore the need for alternative therapeutic strategies. Emerging treatments, such as non-intensive venetoclax-based regimens, aim to address these challenges by offering reduced toxicity and improved tolerability without compromising efficacy (shown in Table 2); however, it is important to highlight that the non-intensive venetoclax-based regimens were developed to treat patients unfit for intensive chemotherapy. The remarkable results shown in the different studies, on one hand, showed that complete remission is achievable and, on the other, showed that ineligibility for intensive chemotherapy does not overlap with ineligibility for allogeneic HSCT.

## 4. Discussion

### Conclusions and Future Perspectives

The treatment landscape for acute myeloid leukemia (AML) has undergone remarkable evolution over the past five decades, moving from an early reliance on intensive chemotherapy to the incorporation of targeted therapies and less intensive options for elderly and unfit patients. While intensive chemotherapy remains a cornerstone for many AML patients, its role in the elderly is increasingly being re-evaluated due to significant limitations in terms of toxicity, tolerability, and long-term outcomes. Elderly patients often face suboptimal results with intensive regimens, including low rates of complete remission (CR) and limited ability to transition to potentially curative allogeneic hematopoietic stem cell transplantation (allo-HSCT) [37,38].

Non-intensive chemotherapy strategies have fundamentally changed the approach to treating older AML patients. Low-dose cytarabine (LDAC) and hypomethylating agents such as azacitidine and decitabine have provided viable alternatives to intensive regimens, offering improved safety profiles but modest efficacy. However, the relatively limited survival benefits and response rates associated with these therapies highlight the need for further innovation in this field.

The emergence of venetoclax-based regimens marks a paradigm shift in the management of AML. The combination of venetoclax with hypomethylating agents (e.g., azacitidine or decitabine) or low-dose cytarabine has demonstrated unprecedented efficacy in elderly and unfit patients. Clinical trials such as VIALE-A and VIALE-C have reported high response rates and meaningful extensions of survival with these regimens, making them a new standard of care for older patients who are unable to tolerate intensive chemotherapy. The VEN-DEC study further underscores the potential of venetoclax-based regimens. By achieving high remission rates (CR/CRi of 74% in VEN-DEC) and enabling a significant proportion of patients to proceed to allo-HSCT (83% in VEN-DEC), this approach not only improves short-term outcomes but also creates a pathway for potentially curative treatment; that study also shows that this therapeutic program can preserve patients’ clinical fitness and serve as a bridge to allo-HSCT. The VEN-DEC findings emphasize that venetoclax-based regimens can serve as an effective bridge to transplantation, a benefit that was historically unattainable for most elderly AML patients undergoing intensive chemotherapy. Furthermore, the VEN-DEC study confirms the safety of this strategy, allowing most patients to continue treatment in an outpatient setting, which could possibility reduce the costs associated with longer hospital admission for intensive chemotherapy.

With the current state of the art, elderly AML patients have several treatment options available. Although the curative role of allo-HSCT is beyond debate, there remains uncertainty about how best to achieve CR and transition as many patients as possible to allo-HSCT. It is crucial to continually reassess the suitability of intensive chemotherapy versus non-intensive strategies across different age groups and types of AML to offer the best and the safest treatment to a such frail population. Notably, all referenced studies report similar rates of CR and induction success (Table 3), and this is the main reason for debate. Recently, non-intensive regimens have demonstrated the ability to control the disease on one hand and to mitigate treatment-related adverse events on the other. Furthermore, increased knowledge regarding the roles of specific somatic gene mutations in patients treated with both intensive and non-intensive therapies has emerged. While some centers have chosen to offer venetoclax-based non-intensive therapy arbitrarily, there is still evidence suggesting efficacy and a relatively high transplant rate in AML patients with either GO-GO or SLOW-GO phenotypes, as demonstrated in a recent French study [39].

Additionally, recent studies have highlighted that patients with mutations in TP53, MECOM, and KRAS exhibit poor prognosis regardless of the induction therapy type or transplantation intensity [40].

**Table 3:** This table synthesizes the principal studies cited in this review, encompassing a wide spectrum of therapeutic approaches for elderly AML patients. The included studies range from traditional intensive chemotherapy regimens (e.g., 7 + 3, FLAG-IDA, CPX-351) to non-intensive and targeted treatments (e.g., VEN-DEC, AZA/IVO, DEC/IBR). For each study, key efficacy endpoints are reported: complete remission (CR) rate, overall survival (OS), and event-free survival (EFS) when available. This table is structured to reflect the evolution of treatment strategies: (1) Intensive chemotherapy regimens (e.g., 7 + 3, CPX-351, FLAG-IDA) show variable CR rates (from 35% to over 70%) but generally limited OS in elderly patients. The CPX-351 studies show some improvement in both CR and OS, particularly in high-risk or adverse cytogenetic subgroups. (2) GIMEMA studies (AML-13, AML-15) focus on feasibility and outcomes of intensive therapy with or without autologous stem cell transplantation in elderly patients, reporting modest survival benefits and underlining the limitations of autologous strategies in this population. (3) Hypomethylating agents plus venetoclax, such as in the VEN-DEC and VIALE-A trials, demonstrate significantly higher CR rates (up to 74%) and improved survival, redefining the standard of care for unfit or older AML patients. (4) Targeted therapy combinations, such as AZA/IVO (AGILE) and DEC/IBR (HOVON135), further expand the therapeutic possibilities, with the AGILE study notably reporting a median OS of 24 months with azacitidine and ivosidenib—one of the highest among non-intensive strategies. (5) Novel investigational approaches, such as CLAD/LDAC alternating with 5-AZA, show promising response rates (68%) and durable remissions, though further validation is needed. Taken together, the data emphasize the shift from traditional chemotherapy toward more personalized and targeted strategies, with better tolerability and improved outcomes for elderly patients who are often ineligible for intensive treatment or transplantation.

Study Name	Study Design	CR Rate	OS (Months)	EFS (Months)
7 + 3 [32]	Study evaluating the activity of higher daurnorubicine doses in elderly AML	High-dose dauno: 52% Standard-dose dauno: 35%	2-year OS, high-dose dauno: 31% 2-year OS, standard-dose dauno: 26%	2-year OS, high-dose dauno: 20% 2-year OS, standard-dose dauno: 17%
CPX-351 [33]	Liposomal combination of Arac-Dauno in high-risk AML versus standard 3 + 7	CPX: 38% 7 + 3: 26%	Median OS CPX: 9.3 Median OS 7 + 3: 6	Median EFS CPX: 5 Median EFS 7 + 3: 3.2
AML 18 [34]	Treatment intensification based on residual disease	CR rate: 70%	15	Not available
AML19 [35]	CPX-351 vs. FLAG-IDA in AML with adverse karyotypes	CPX: 64% FLAG-IDA: 73%	Median OS CPX: 13.3 Median OS FLAG-IDA: 11.4	Median EFS CPX: 22.1 Median EFS FLAG-IDA: 8.35
GIMEMA AML-13 [20]	Phase III trial evaluating intensive chemotherapy in elderly patients and autologous stem cell transplantation as consolidation	50%	18	12
GIMEMA AML-15 [19]	Phase II study assessing intensive chemo in elderly patients	CR+CRi 50%	9.4	Not available
VEN-DEC [29]	Phase II trial of venetoclax + decitabine in elderly AML patients	74%	Median OS: not reached	Median EFS: not reached
VIALE-A [28]	Phase III prospective randomized study—AZA/VEN vs. AZA/PCB	48% vs. 13%	14.7 vs. 9.6	9.8 vs. 7
VIALE-C [24]	Phase III prospective randomized study—LDAC/VEN vs. LDAC/PCB	34% vs. 3%	8.4 vs. 4.1	4.7 vs. 2
AGILE [11]	Phase III prospective randomized study—AZA/IVO vs. AZA/PCB	47% vs. 11%	24 vs. 7.9	22.9 vs. 4.1
HOVON135 [36]	Phase III prospective randomized study—DEC/IBR vs. DEC	50% vs. 41%	11.5 vs. 11	Median: 13
CLAD/LDAC alternating with 5-AZA [41]	Phase II prospective study	68%	13.8	Not reached
NCRI AML 14 [23]	Prospective randomized trial—LDAC vs. HU	LDAC: 18%	Less than 12 months for both arms	Less than 12 months for both arms
DACO-016 [42]	Prospective randomized phase III study—decitabine vs. LDAC or BAT	DEC: 15.7% LDAC/BAT: 7.9%/3.6%	Median OS DEC: 7.7 Median OS LDAC/BAT: 5	Median PFS DEC: not available Median PFS LDAC/BAT: not available
AZA-AML-001 [27]	Prospective randomized phase III study—azacitidine vs. conventional care regimens	AZA: 27.8% CCR: 25.1%	Median OS AZA: 10.4 Median OS CCR: 6.5	Median PFS AZA: 6.7 Median PFS CCR: 4.8

## 5. Conclusions

The representativeness of the studies cited in this review has been carefully considered to encompass a broad spectrum of clinical scenarios and patient demographics. However, it is crucial to acknowledge that while this selection aims to reflect general trends and outcomes, individual study characteristics, such as patient selection criteria and baseline characteristics, may have limited the generalizability of the results. Also, as a narrative comparative synthesis, it does not include a formal meta-analysis or risk-of-bias assessment, which limits the quantitative integration of the results. The included studies are heterogeneous in terms of design, patient characteristics, and outcome definitions, which may affect the generalizability of the conclusions. Moreover, while the selection of the featured studies was based on clinical relevance and impact within the field, the absence of a systematic protocol may have limited reproducibility. Future studies should aim to include more diverse populations to validate the efficacy and safety of these treatments across different subgroups. Furthermore, the discussion of the long-term implications of non-intensive therapies remains paramount. Although these therapies offer significant improvement in tolerability and initial response rates, their long-term impacts on survival and quality of life need further exploration. Notably, while venetoclax-based regimens show promise in bridging patients to potentially curative therapies like allo-HSCT, the sustainability of remission and post-transplantation outcomes require more extensive longitudinal studies. This highlights the necessity for ongoing research to optimize these regimens, mitigate potential long-term adverse effects, and enhance overall patient outcomes in the context of chronic disease management.

Looking forward, the integration of venetoclax-based regimens into non-intensive AML treatment protocols has set the stage for further advancements. Future research should aim to:

1. Optimize venetoclax dosing and schedules to minimize toxicity while maintaining efficacy;
2. Identify predictive biomarkers for responses to venetoclax-based therapies to better personalize treatment;
3. Investigate novel combinations of venetoclax with emerging targeted agents, such as FLT3 inhibitors, IDH1/2 inhibitors, and immunotherapies, to enhance efficacy across diverse AML subtypes;
4. Develop strategies to reduce post-transplantation relapse rates for patients who transition to allo-HSCT following venetoclax-based induction.

With continued innovation, it is conceivable that the historically poor prognosis for older AML patients may be significantly improved in the coming years, providing hope for a population that has long been underserved in hematological oncology.

## Figures and Tables

**Table 1:** The main studies of the treatment of acute myeloid leukemia (AML) in the elderly (age ≥ 60 years) and the corresponding rates of allogeneic transplantation in first complete remission (CR1). This table summarizes the main clinical trials that have evaluated therapeutic strategies in elderly patients with AML, with particular focus on complete remission (CR) rates and the proportion of patients undergoing allogeneic stem cell transplantation in CR1. It includes studies on both traditional intensive chemotherapy and liposomal formulations (such as CPX-351), as well as innovative approaches based on venetoclax combined with hypomethylating agents. This table highlights the evolution of treatment strategies and the increasing role of transplantation in this age group, as notably demonstrated by the VEN-DEC study, which reported a transplantation rate of 83% among the responding patients.

Study Name	Study Design	Results and CR Rate	Proportion of Patients Allografted in CR1
7 + 3 [32]	Study evaluating the activity of higher daurnorubicine doses in elderly AML	High dose dauno: 52% Standard dose dauno: 35%	11%
CPX-351 [33]	Liposomal combination of Arac-Dauno in high-risk AML versus standard 3 + 7	CPX: 38% 7 + 3: 26%	26%
AML 18 [34]	Treatment intensification based on residual disease	CR rate: 70%	36%
AML 19 [35]	CPX-351 vs. FLAG-IDA in AML with adverse karyotypes	CPX: 64% FLAG-IDA: 73%	39%
GIMEMA AML-13	Phase III trial evaluating intensive chemotherapy in elderly patients and autologous stem cell transplantation as consolidation	50%	0%
GIMEMA AML-15	Phase II study assessing intensive chemo in elderly patients.	CR+CRi 50% Median OS < 1 year	0%
VEN-DEC	Phase II trial of venetoclax + decitabine in elderly AML patients	74%	83%

**Table 2:** Non-intensive chemotherapy regimen options for the treatment of elderly AML patients. This table summarizes key randomized clinical trials evaluating non-intensive chemotherapy regimens in elderly patients with acute myeloid leukemia (AML), comparing experimental combinations to standard therapies. The reported outcomes include complete remission (CR) rates, overall survival (OS), and event-free survival (EFS). The results highlight the improved efficacy of novel combinations—particularly those including venetoclax (VEN) and targeted agents such as ivosidenib or ibrutinib—over conventional regimens with hypomethylating agents or low-dose cytarabine. Notably, the AGILE study demonstrated a marked improvement in overall survival (24 vs. 7.9 months) with azacitidine plus ivosidenib compared to the control arm.

Study Name	Study Design	CR Rate	OS (Months)	EFS (Months)
VIALE-A [28]	Prospective randomized AZA/VEN vs. AZA/PCB	48% vs. 13%	14.7 vs. 9.6	9.8 vs. 7
VIALE-C [24]	Prospective randomized LDAC/VEN vs. LDAC/PCB	34% vs. 3%	8.4 vs. 4.1	4.7 vs. 2
AGILE [11]	Prospective randomized AZA/IVO vs. AZA/PCB	47% vs. 11%	24 vs. 7.9	22.9 vs. 4.1
HOVON135 [36]	Prospective randomized DEC/IBR vs. DEC	50% vs. 41%	11.5 vs. 11	Median 13

## Data Availability

Not applicable.
